# Draft Genome Sequence of Bacillus thuringiensis SS2, from Dibenzofuran-Contaminated Soil, Reveals Putative Aromatic Compound Degradation Genes

**DOI:** 10.1128/mra.00198-23

**Published:** 2023-04-26

**Authors:** Salametu Saibu, Imade Y. Nsa, Ayodeji A. Odunsi, Nwanneka M. Akinyemi, Ganiyu O. Oyetibo, Sunday A. Adebusoye

**Affiliations:** a Department of Microbiology, Faculty of Science, University of Lagos, Lagos, Nigeria; b Department of Microbiology, Faculty of Science, Lagos State University, Lagos, Nigeria; University of Maryland School of Medicine

## Abstract

The draft genome sequence of strain Bacillus thuringiensis SS2 consists of 426 contigs assembled at the scaffold level, totaling 5,030,306 bp, and contains 5,288 putative PATRIC protein-coding genes, including genes responsible for total benzoate consumption, degradation of halogenated compounds, heavy metal tolerance/resistance, biosynthesis of secondary metabolites, and microcin C7 self-immunity protein.

## ANNOUNCEMENT

Bacillus thuringiensis is a Gram-positive, aerobic, rod-shaped, mesophilic bacterium that has been reported to metabolize persistent organic pollutants (POPs), pesticides, and heavy metals (1, 2). B. thuringiensis SS2 was identified during a screen for dioxin-degrading bacteria from dibenzofuran (DF)-polluted waste dump soil obtained with a site with a history of incineration in Cele, Isolo, Lagos, Nigeria (3).

B. thuringiensis SS2 was isolated by an enrichment procedure over a 12-month period, in a chloride-free minimal salts medium inoculated with dioxin-polluted soil in the presence of dioxin congeners at 28 ± 2°C with agitation; it could utilize DF, 2,8-dichlorodibenzofuran (2,8-diCDF), and 2,7-dichlorodibenzo-*p*-dioxin (2,7-diCDD) (3). It was identified using 16S rRNA gene sequencing (GenBank accession number MH714859) and biochemical characterization (3).

The genomic DNA of B. thuringiensis SS2 was extracted using the Quick-DNA fungal/bacterial miniprep kit (Zymo Research, Irvine, CA, USA), from a Luria-Bertani culture that had been grown overnight at 28°C. The DNA was quantified using a QuantiFluor double-stranded DNA (dsDNA) system with a Qubit 3.0 fluorometer. Whole-genome sequencing (WGS) was performed through Illumina NextSeq paired-end sequencing. The library preparation and 2 × 150-bp paired-end sequencing were performed using the Nextera XT Index kit v2 (FC-131-2001 to FC-131-2004) and a NextSeq 500 system (Illumina, San Diego, CA, USA) at Quadram Institute Bioscience (Norwich, UK) (4).

The Trim Galore tool v0.6.5 (5) was used for preassembly trimming, and the quality of the reads was assessed using QUAST v5.0.2 (6, 7). The number of reads generated was 4,149,337, with an average length of 146 bp. Genome assembly was performed using Unicycler v0.4.8 (8), and the assembled contigs were annotated using RASTtk v1.073 (9) at PATRIC (now Bacterial and Viral Bioinformatics Resource Center [BV-BRC]) v3.28.9 (10). All tools were run on PATRIC (BV-BRC) with default parameters.

The genome sequence of B. thuringiensis SS2 comprised a total of 5,030,306 bp (sequencing depth, 228.091×), forming 426 contigs (GC content, 35.67%). The longest contig was 120,678 bp, with an *N*_50_ value of 22,410 bp. RASTtk identified 5,288 PATRIC protein-coding sequences and 1,174 hypothetical proteins, 66 tRNA genes, and 4 rRNA genes; the closest relative for B. thuringiensis SS2 is B. thuringiensis 97-27 (serovar konkukian).

WGS of B. thuringiensis SS2 revealed putative genes linked to the degradation of dioxin and other POPs similar to those of Serratia marcescens SSA1, which was isolated from the same burnt dump site. These genes include putative genes for total consumption of benzoate via hydroxylation and halogenated aromatic compound (biphenyl, toluene, xylene, styrene, benzoate, atrazine, and chloroethene) degradation, among others (4). B. thuringiensis SS2 contains putative lead-, cadmium-, zinc-, and mercury-transporting ATPase (EC 3.6.3.3 and EC 3.6.3.5) and copper-translocating P-type ATPase (EC 3.6.3.4) genes, suggesting heavy metal tolerance and resistance. Other putative genes, including those for microcin C7 self-immunity protein (*mccf*), macrocin *O*-methyltransferase, and CAAX amino-terminal proteins, could indicate this strain’s diverse metabolic adaptations to stress in its environment.

### Data availability.

The genome annotation of B. thuringiensis SS2 at PATRICbrc (BV-BRC) can be viewed at https://www.bv-brc.org/view/Genome/1428.1921. The draft genome sequence was deposited in GenBank under the accession number JAOWLZ000000000; project data are available under BioProject accession number PRJNA888024, with BioSample accession number SAMN31205655 and SRA accession number SRR21870348. The 16S rRNA gene sequence is available under GenBank accession number MH714859.
